# Establishment and Partial Characterization of Canine Mammary Tumor Cell Lines

**DOI:** 10.3390/ani15131991

**Published:** 2025-07-07

**Authors:** Eliza Vazquez, Luis Dominguez, Brian Silverio, Geobanni Torres, Adriana Garibay-Escobar, Felisbina Luisa Queiroga, Carlos Velazquez

**Affiliations:** 1 Department of Chemistry-Biology, University of Sonora, Blvd. Luis Encinas y Rosales s/n, Hermosillo 83000, Mexico; eliza.vazquez@unison.mx (E.V.); a214206530@unison.mx (L.D.); a217217239@unison.mx (B.S.); a210206416@unison.mx (G.T.); adriana.garibay@unison.mx (A.G.-E.); 2 Veterinary and Animal Research Centre (CECAV), University of Trás-os-Montes and Alto Douro, 5001-801 Vila Real, Portugal

**Keywords:** cancer, canine mammary tumors, cell lines, breast cancer, cell culture

## Abstract

Mammary tumors are the most common neoplasms diagnosed in female dogs and have been considered excellent models for studying human breast cancer. Establishing cell lines from primary cultures of canine mammary tumors provides an in vitro model to better understand the disease and develop new treatments. This study aimed to establish and characterize canine mammary tumor cell lines. Ten cell cultures were generated from tumor tissue obtained from affected dogs, including seven from primary mammary tumors and three from metastatic sites. Characterization included molecular marker expression (ER, PR, HER2, cytokeratin 5/6 (CK5/6), vimentin, and the marker of cell proliferation Ki67) and in vitro tumorigenic capacity assessment. Additionally, the susceptibility of five cell lines to DOX, 5-FU, paclitaxel, colchicine, and carboplatin was evaluated using the MTT assay. ICC analysis revealed negative expression of hormonal receptors (ER and PR) in five cell lines, while only one cell line was positive for both. Six cell lines were HER2-negative and positive for vimentin. Five cell lines exhibited in vitro tumorigenic capacity, forming colonies in soft agar. DOX showed the highest growth-inhibitory effect (DOX > Paclitaxel > Colchicine > 5-FU > Carboplatin). Two cell lines had a minimal concentration for 50% inhibition in vitro (IC_50_) < 0.63 µM and 4.37 ± 0.40 µM for DOX, while one was sensitive to colchicine and paclitaxel (IC_50_ 0.19 µM and 0.04 µM, respectively). All tested cell lines were resistant to carboplatin and 5-FU. These cell lines provide a valuable model for studying breast cancer in humans and dogs and evaluating new potential therapeutic strategies.

## Simple Summary

Mammary tumors are the most frequent neoplasia in female dogs. Canine mammary cell lines represent a model of study for women breast cancer research that could benefit both species. The aim of our study was to establish and partially characterize canine mammary tumor cell lines. Ten cell cultures were generated from tumor tissue obtained from canines with mammary tumors. Seven cell lines were generated from primary mammary tumors and three cell lines from metastatic sites. Immunocytochemistry (ICC) analysis revealed a negative expression of hormonal receptors (estrogen receptor [ER] and progesterone receptor [PR]) in five cell lines and positive in one cell line, whereas six cell lines were human epidermal growth factor receptor 2 (HER2)-negative and positive for vimentin. Several cell lines showed tumorigenicity capacity in vitro. The susceptibility of five cell lines to different drugs was evaluated with the MTT (3-(4,5-dimethylthiazol-2-yl)-2,5-diphenyltetrazolium bromide) assay, where doxorubicin (DOX) showed the highest growth-inhibitory effect (DOX > Paclitaxel > Colchicine > 5-Fluorouracil [5-FU] > Carboplatin). The cell lines generated represent a model of study for breast cancer that can be used for developing and testing new treatments to improve the survival of canine and human patients.

## 1. Introduction

Cancer represents the leading cause of mortality in dogs over the age of ten, with approximately 50% of these animals developing neoplastic diseases and one in four succumbing to them [1]. Notably, dogs develop spontaneous tumors that mirror human cancers in their biological behavior, clinical manifestations, pathological features, and underlying molecular mechanisms [2,3,4]. Among these, mammary tumors are the most frequently diagnosed neoplasms in both female dogs and women, affecting a wide range of mammalian species and constituting a major concern in both veterinary and human public health [5,6]. Understanding the presentation and progression of breast cancer across different species enhances our knowledge of the disease’s complex pathogenesis [7]. Beyond serving as sentinels for environmental and lifestyle-associated carcinogenic exposures, tumor-bearing dogs are increasingly recognized as translational models for drug development, therapeutic innovation, and preclinical trials [3,7,8,9,10,11].

In both dogs and humans, the incidence of mammary tumors is highly variable, and impacted by biological, pathological, cultural, and socioeconomic factors, including hormonal status, breed, advanced age, obesity, diet, among other factors [12,13,14,15]. Similarities of breast cancer between humans and dogs have also been identified at the molecular level, involving the overexpression of steroid hormone receptors, such as estrogen (ER) and progesterone (PR), proliferation markers (Ki67, AgNOR), epidermal growth factor (EGF), and cyclooxygenases, among others [8,16,17,18,19,20,21].

Canine cell lines derived from mammary tumors represent a relevant in vitro model for studying breast cancer, facilitating a deeper understanding of the disease and the development of receptor-targeted therapies. These models are also crucial for preclinical drug studies. Previous studies have established and partially characterized around 60 canine mammary cell lines, most of them from mammary tumors, and a few from metastatic sites and from inflammatory carcinoma [4,22,23,24,25,26,27,28,29,30,31,32,33,34,35,36,37,38,39,40]. Canine mammary cell lines were generated in different countries such as China, Korea, Japan, Turkey, Spain, Scotland, Sweden, Netherlands, Taiwan, and Iran, but in Latin America, according to the available literature, Brazil is the only country that had published canine cell lines [30]. In the present study, the tumor cell lines were obtained from dogs living in Mexico. In dogs, it remains unclear if the breed or the geographical region has an effect on the behavior, outcome, or response to treatments of mammary tumors, as reported in women’s breast cancer [41]. Also, canines are considered as sentinels for human exposures to chemicals, herbicides, infectious diseases, environmental hazards, among others, that can play a role in the development of cancer [42,43,44].

Despite their importance, the availability of canine breast cancer cell lines remains relatively limited. For example, the American Type Culture Collection (https://genomes.atcc.org/, accessed on 23 January 2025), a private nonprofit organization that serves as a biological resource center, holds only two cell lines from canine mammary tumors. Similarly, the European Collection of Authenticated Cell Cultures (https://www.culturecollections.org.uk/, accessed on 23 January 2025), which houses authenticated cell cultures from around the world, also has only two cell lines from canine mammary tumors. This limited availability poses a challenge for comprehensive research and therapeutic development [4,30,31,33,35,36,37,38,45].

The main aim of the present study was to establish and perform a partial characterization of canine mammary cell lines obtained from both tumor and metastatic sites of different histological subtypes in dogs living in Mexico.

## 2. Materials and Methods

### 2.1. Tumor Samples

**Figure 1:**
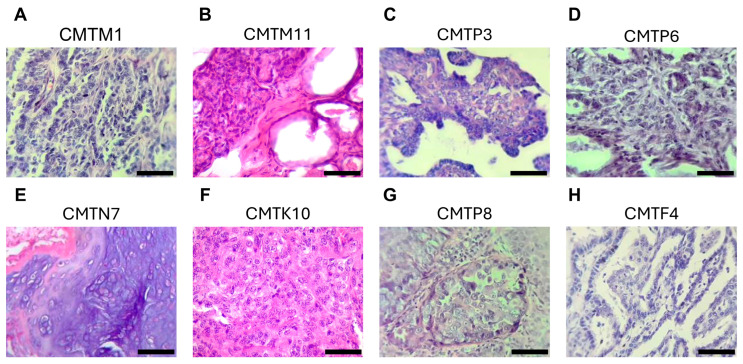
Histopathological classification of primary canine mammary tumors. (**A**–**H**) Tumor paraffin sections, stained with hematoxylin and eosin. (Scale bar = 50 μm).

The study was submitted to the Research Ethics Committee of the University of Sonora (CEI-UNISON) under the project ID USO313007546. Seven tumor samples of approximately 1 cm^3^ were sterilely collected after the surgical removal for diagnosis and therapeutic purposes from dogs with mammary gland tumors (Table 1). The rest of the tumor was fixed in formalin and embedded in paraffin for histopathological examination, which was conducted by a board-certified veterinary pathologist following the criteria established by Goldschmidt et al. (2011) [46]. This classification includes both histotype identification and malignancy grading based on cellular morphology, tubule formation, nuclear pleomorphism, and mitotic count (Figure 1). All dogs were admitted to two referring canine hospitals in Hermosillo, Sonora, Mexico. Tissue samples were provided with permission from the owners, and a previous explanation of the protocol and informed consent were signed. Tumor samples were transported to laboratory in sterile conical tubes with Dulbecco’s Modified Eagle’s medium (DMEM) containing 10% fetal bovine serum (FBS, Gibco, Thermo Fisher Scientific, Waltham, MA, USA), supplemented with antibiotics (penicillin 100 IU/mL and streptomycin 100 IU/mL) (D10F) at 4 °C. Three samples from neoplastic pleural effusions were collected from dogs diagnosed with mammary tumors after thoracocentesis. The effusion samples were transported in sterile tube at 4 °C. Biological samples were processed within 2–3 h of collection.

### 2.2. Establishment of Primary Cultures from Canine Mammary Tumors

Tumor samples were collected immediately after surgical excision and transported in D10F medium under sterile conditions. Each sample was placed in a sterile Petri dish with 2 mL of D10F and minced into 3–4 mm pieces using sterile scissors and a scalpel, carefully removing connective and necrotic tissue. The fragments were transferred to a 15 mL conical tube containing 0.5 mL of collagenase (200 U/mL; C0130, Sigma-Aldrich, St. Louis, MO, USA), 0.5 mL of hyaluronidase (100 U/mL; H3506, Sigma-Aldrich, St. Louis, MO, USA), and 6 mL of D10F. The tubes were incubated overnight at 37 °C in a humidified 5% CO_2_ atmosphere with continuous rotation (360°).

Following digestion, the mixture was centrifuged at 600× *g* for 5 min. The supernatant was discarded, and the pellet resuspended in fresh D10F. The suspension was filtered through a sterile cell strainer into a 50 mL conical tube, adjusted to 5 mL, and seeded into a 25 cm^2^ tissue culture flask. Cultures were incubated at 37 °C in a humidified 5% CO_2_ incubator (Isotemp, Thermo Fisher Scientific, Waltham, MA, USA), and medium was changed three times per week.

**Figure 2:**
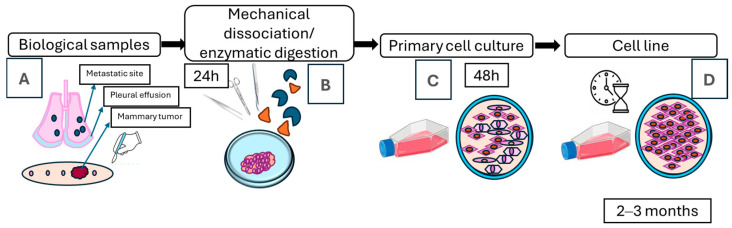
Workflow of cell line generation from different biological samples of canines with mammary tumors. (**A**) Obtention of biological sample, (**B**) mechanical dissociation and/or enzymatic digestion, (**C**) generation of primary cell culture at 48 h, visualizing different cell morphology, small pieces of tissue known as organoids, (**D**) followed by subcultures for 2–3 months until the generation of cell line.

After 24–48 h, adherent cells or tissue fragments began to attach. Once confluence reached approximately 80–90% (typically after 7 days), the cultures were passaged using 0.25% trypsin solution (Gibco, Thermo Fisher Scientific, Waltham, MA, USA) and subcultured into new flasks with fresh medium. This process was repeated at least 20 times over 3–4 weeks. The cultures were considered established cell lines when they showed consistent morphological characteristics, stable proliferation, and capacity for cryopreservation and recovery. Cells were frozen in medium containing 10% of dimethyl sulfoxide (DMSO) and stored at −150 °C (Figure 2) [47,48].

Pleural effusion samples were processed without enzymatic digestion. The fluid was centrifuged at 500× *g* for 7 min at 4 °C, the supernatant discarded, and the pellet resuspended in D10F and transferred to a 25 cm^2^ flask. Cultures were monitored daily, and confluent monolayers (80–90%) were typically observed after 3 days. Cells were passaged using 0.25% trypsin and subcultured for at least 20 passages. These cultures were also cryopreserved at −150 °C.

### 2.3. Cell Doubling Time

Cells were seeded into 12-well culture plates (30,000 cells/well) in D10F. Confluent cell cultures were trypsinized and counted every 24 h for 6 days. All measurements were carried out in duplicate. A cell growth curve was established to identify the log phase, and the cell doubling time (DT) was calculated using the following equation [47,49]:

DT = h/[ln(N_2_/N_1_)/ln(2)]

(N_2_ indicates the number of cells counted at time, h; N_1_ indicates the number of cells seeded). All experiments were performed in triplicate.

### 2.4. Soft Agar Assay

The colony-forming efficiency was determined by using a soft agar assay previously described [50]. This in vitro assay evaluates one of the hallmarks of cancer cells, which is the anchorage-independent growth. The bottom layer was prepared with equal volumes of 1% agarose (Sigma-Aldrich, A9539, St. Louis, MO, USA) and 2× D10F (medium prepared using double amounts of the ingredients); 2 mL of this solution was poured into each well of a 6-well culture plate. The base agar layer was allowed to solidify for 5–10 min. The second layer was prepared with a mixture of 0.7% agarose, cell suspension with 2× D10F in a 1:1 ratio, and 2 mL of this suspension was added quickly to the base agar layer. Each well contained 100,000 cells. The agarose was allowed to solidify for 5–10 min, and 2 mL of D10F was added on the top of each well. The cell cultures were seeded in triplicate and incubated at 37 °C with 5% CO_2_ for 3 weeks, and the medium was changed twice a week. The colony growth was observed by using an inverted microscope. MCF7 cell line was used as a positive control of colony formation capacity [50,51,52].

### 2.5. Immunocytochemistry

An immunocytochemistry (ICC) study was performed using primary antibodies against ER (1:100, mouse, TE111.5D11, Invitrogen, Waltham, MA, USA), PR (1:100, mouse, PR-AT.14, Invitrogen, Waltham, MA, USA), cytokeratin 5/6 (CK5/6) (1:100, mouse, D5/I6B4, Invitrogen, Waltham, MA, USA), vimentin (1:100, mouse, O91D3, BioLegend, San Diego, CA, USA), human epidermal growth factor receptor 2 (HER2) (1:100, rabbit, SP3, Invitrogen, Waltham, MA, USA), and Ki67 (1:100, mouse, K2, Leica Biosystem, Deer Park, IL, USA). All antibodies used in this study have demonstrated cross-reactivity with canine antigens, as reported by the manufacturers and supported by previously published studies in canine mammary tumors [53,54,55,56,57,58]. Commercial cell lines MCF7, T47D, SKBR-3, and MDA-MB-231 cell lines were used as positive controls for the expression of ER, PR, HER2, and Ki67 molecules, respectively. Appendix tissue was used as positive control for vimentin expression, and epidermis with hair follicles tissue was the positive control for CK5/6.

Cells were cultured on coverslips placed in 6-well plates using D10F medium until confluence was attained. Once confluent, cells were rinsed twice with PBS and fixed in ethanol at −20 °C for 20 min. After fixation, they were washed twice with PBS and incubated for 15 min with 1% bovine serum albumin (BSA) in PBS to block non-specific binding. Following another two PBS washes, peroxidase blocking solution was applied for 5 min and then removed by rinsing with PBS. The primary antibody was incubated for 50 min, followed by three PBS washes. Next, the secondary antibody (Mouse Probe, MACH1, Biocare, Pacheco, CA, USA) was applied and incubated at room temperature for 20 min. After three additional PBS washes, the tertiary antibody (HRP-polymer, MACH1, Biocare, Pacheco, CA, USA) was added and incubated for 30 min. Cells were then washed again and incubated with 3,3′-Diaminobenzidine (DAB) for 10 min, followed by rinsing with distilled water. A brief counterstaining with Harris hematoxylin (HHS32, Sigma-Aldrich, St. Louis, MO, USA) was performed for 10 s, then the cells were rinsed again with distilled water. Finally, the coverslips were mounted with resin for microscopic observation.

Each marker was evaluated by comparison with the corresponding positive and isotype controls. For ER and PR, cases were considered positive when more than 1% of tumor cells showed nuclear staining. HER2 expression was scored semiquantitatively as follows: 0 (no staining), 1+ (weak, incomplete membranous staining in <10% of cells), 2+ (weak to moderate complete membranous staining in >10% of cells), and 3+ (strong complete membranous staining in >10% of cells); only 3+ was considered positive. Ki67 expression was recorded as the percentage of tumor cells showing nuclear positivity, ranging from 1–100%. The analysis was performed across the entire area of the coverslip (625 mm^2^) by two qualified analysts, following the guidelines of Peña et al. (2013) [59].

### 2.6. Evaluation of Susceptibility of Cell Lines to Different Cancer Drugs

Determination of susceptibility of cell lines to different drugs was evaluated by MTT (3-(4,5-dimethylthiazol-2-yl)-2,5-diphenyltetrazolium bromide) assay in accordance with the standard protocol [60]. This assay is a colorimetric method to determine the effect or cytotoxicity of drugs on the cell culture and is based on the reduction of yellow MTT into purple formazan by metabolically active cells. In 96-well cell culture plates (Costar, Corning, NY, USA), 10–20 × 10^3^ cells (50 µL) were seeded. The cells were incubated for 24 h at 37 °C with 5% of CO_2_ to allow adhesion. Cells were treated with different concentrations (50 µL) of the following drugs: Doxorubicin (DOX), 5-Fluorouracil (5-FU), colchicine, paclitaxel, and carboplatin; DMSO and PBS were used as controls. Cells were incubated for 48 h. In the last 4 h, the treatments were removed, and cells were washed with PBS; 100 µL of D10F and 10 µL of MTT solution (5 mg/mL) were added to each well. After that, 100 µL of acidified isopropanol was used to dissolve the formazan crystal. The optical density was measured with a plate reader (Multiskan Go, ThermoLab System, Waltham, MA, USA) at 570–630 nm. The antiproliferative activity of the drugs was reported as IC_50_ values (IC_50_ was defined as the required concentration of the drug to inhibit cell proliferation by 50%). At least three independent experiments were performed in triplicate for each cell line.

## 3. Results

### 3.1. Establishment of Cell Lines from Canine Mammary Tumors

Cell lines were successfully established from a total of 10 biological samples, including 3 from pleural effusions of metastatic dogs and 7 from mammary tumors of different female dogs. Of the mammary tumor-derived cell lines, 3 were from benign tumors as determined by histopathological analysis: CMTM1, characterized as an intraductal papillary adenoma; CMTM11, identified as a tubular adenoma with cellular dysplasia; and CMTP3, a benign mixed tumor with intraductal papillary adenoma features. Additionally, 3 of the cell lines originated from malignant tumors: CMTP6 (tubular carcinoma), CMTN7 (mixed-type adenocarcinoma), and CMTK10 (solid adenocarcinoma). The CMTL9 cell line was established from a mammary tumor specimen lacking histopathological characterization. It originated from a canine patient presenting with multiple mammary tumors, among which the largest lesion measured approximately 3 cm, exhibited rapid growth, and displayed a hemorrhagic region. Additionally, the inguinal lymph node was notably enlarged, suggesting potential metastatic involvement. The remaining three cell lines, established from metastatic sources, included CMTG5 from pleural effusion associated with infiltrating tubulopapillary carcinoma, CMTP8 from pleural effusion linked to intraductal carcinoma, and CMTF4 from a lung metastasis and pleural effusion related to infiltrating intraductal papillary carcinoma. Table 1 summarizes the clinical characteristics of the canine patients from which the mammary tumor cell lines were established, while Figure 1 shows the histopathological characteristics of the corresponding tumors.

Tumor samples underwent mechanical and enzymatic digestion. The passages continued for 2–3 months (Figure 2); during this period, the primary cell cultures showed diverse cell morphologies. After 3–4 weeks, the cell cultures showed a homogenous cell morphology. Morphological analysis revealed a predominance of polygonal or “stellate” cells that grew as monolayers, suggestive of an epithelial origin. These cells displayed distinct malignant characteristics, including variation in size, shape, and nuclear pleomorphism. Frequently, the cells exhibited multiple nuclei (up to four), prominent nucleoli (ranging from two to four), and chromatin alterations. Cell lines generated from benign tumors grew slower (>10 days to reach confluency in 25 cm^2^ flask culture) compared to those derived from malignant tumors (which took 7–10 days in a 25 cm^2^ flask culture). Additionally, the cell line generated from pleural effusion exhibited the same malignant cellular morphology (80–90% of cell population) within 2 to 3 days of cell culture. The culture conditions used favored the elimination of contaminating cells.

### 3.2. Doubling Time Determination

The DT was determined for all cell lines generated from metastatic samples (CMTF4, CMTP8, and CMTG5) and in three from mammary tumors (CMTK10, CMTN7, and CMTL9). The cell line CMTK10 had the lowest DT (18 h); in contrast, the cell line CMTG5 showed the highest DT (41 h). DTs: CMTK10 (18 h), CMTF4 (22 h), CMTN7 (23 h), CMTL9 (24 h), CMTP8 (30 h), and CMTG5 (41 h).

### 3.3. In Vitro Tumorigenicity

**Figure 3:**
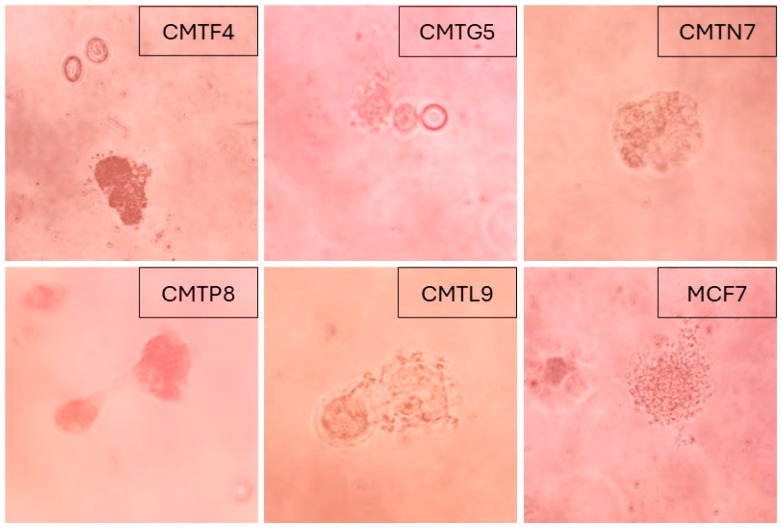
In vitro tumorigenic capacity of the cell lines CMTF4, CMTG5, CMTN7, CMTP8, and CMTL9. (400×). Soft agar colony formation assay was used to evaluate the capability of the cell lines to grow and form colonies in suspension. MCF7 cell line was used as a positive control for colony formation. Images were obtained on day 30.

Anchorage-independent growth was evaluated using the soft agar colony formation assay. CMTP8, CMTG5, CMTF4, CMTN7, and CMTL9 demonstrated the capacity for colony formation, indicating their ability to grow and divide with anchorage-independent potential. CMTP8 formed colonies on day 4, similar to the MCF7 cell line (a human breast cancer cell line used as a positive control). Colonies of the CMTG5 cell line were visible on day 20, while colonies from CMTF4, CMTN7, and CMTL9 cultures became visible on day 30 (Figure 3).

### 3.4. Immunocytochemistry Characterization of Cell Lines

Molecular classification in human breast cancer has been widely applied to determine prognosis and treatment [61]. To determine and classify the molecular phenotype of the canine tumor cell lines, the expression of hormonal receptors, like ER and PR, HER2, and Ki67 (as a proliferation marker) was determined by an ICC analysis. At this time of the investigation, CMTF4, CMTG5, CMTN7, CMTP8, CMTL9, and CMTK10 were selected for this evaluation due to their doubling time and origin; the rest of the cell lines will be analyzed in future experiments. CMTF4, CMTN7, CMTL9, CMTG5, and CMTK10 cell lines exhibited negative expressions for ER, PR and HER2. These results indicate that those cell lines can be considered triple-negative according to human classification [62]. CMTP8 showed positivity for ER and PR but did not express the HER2 and Ki67 markers. These findings suggest that this case could be classified as a luminal A tumor, following the human-based immunohistochemical classification proposed for canine mammary tumors [10,59]. The cell line CMTF4 was Ki67-positive (65%). In contrast, cell lines CMTN7, CMTL9, CMTP8, CMTK10, and CMTG5 did not express the proliferation marker Ki67.

Vimentin is an intermediate filament protein associated with various cellular processes, including migration, differentiation, proliferation, adhesion, invasion, and epithelial–mesenchymal transition (EMT). The cell lines CMTF4, CMTN7, CMTL9, CMTP8, CMTG5, and CMTK10 tested positive for vimentin.

**Figure 4:**
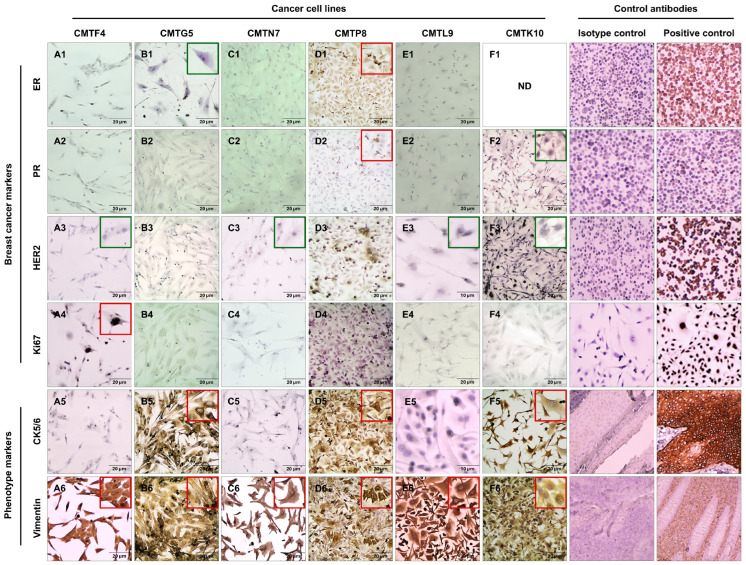
Immunocytochemistry results and morphological analysis of canine mammary cancer cell lines. In the figure, rows represent the cell markers used, and columns correspond to the analyzed cell lines (**A1**–**F6**). Some images include insets in the upper right corner: red-framed insets show close-ups of positive cells, while green-framed insets highlight cellular morphology. Isotype and positive controls: ER—MCF7, PR—T47D, HER2—SKBR-3, Ki67—MDA-MB-231, CK5/6—skin tissue, vimentin—appendix tissue. ND: Not determined.

Cytokeratins are an intermediate filament protein and a component of cell cytoskeleton. CK5/6 is a specific type of cytokeratin primarily expressed in myoepithelial cells and commonly used as a basal epithelial marker. Among the cell lines tested, CMTP8, CMTG5, and CMTK10 showed positive expression for CK5/6; in contrast, the CMTF4, CMTN7, and CMTL9 were negative for this marker (Table 2 and Figure 4).

The CMTF4 cell line (A1–A6) exhibited a Ki67+ (A4) and vimentin+ (A6) immunophenotype. Its morphology (A3) featured polygonal, elongated, and stellate cells, with marked nuclear pleomorphism and the presence of 1 to 3 nucleoli. The CMTG5 cell line (B1–B6) showed a CK5/6+ (B5) and vimentin+ (B6) immunophenotype. In B1, a triangular and elongated cell is observed, with a prominent nucleolus but no nuclear pleomorphism. The CMTN7 cell line (C1–C6) was vimentin+ (C6) and displayed polygonal, elongated, and stellate cells. In C3, a cell with two prominent nucleoli is highlighted. The CMTP8 cell line (D1–D6) exhibited a unique immunophenotype: ER+ (D1), PR+ (D2), CK5/6+ (D5), and vimentin+ (D6). Its cells were polygonal, short, and stellate, with evident nuclear pleomorphism, variations in nuclear size and shape, and the presence of 1 to 2 nucleoli. The CMTL9 cell line (E1–E6) was vimentin+ (E6) and displayed large, short bipolar cells (E3) and polygonal stellate cells (E6). The nuclei exhibited marked pleomorphism, with multinucleated cells (4–8 nuclei) and prominent nucleoli. Finally, the CMTK10 cell line (F1–F6) showed CK5/6+ (F5) and vimentin+ (F6) immunophenotypes. Along with CMTL9, it exhibited the most alterations, including large, elongated, bipolar cells (F2) and polygonal stellate cells (F3). It also displayed severe nuclear pleomorphism, with multinucleated cells (4–8 nuclei) and prominent nucleoli. All cell lines showed morphological features consistent with epithelial cells. Additionally, numerous alterations associated with malignancy were observed, underscoring their utility in canine mammary cancer studies. We used several cancer cell lines and normal tissues as control cells expressing different cellular markers. MCF7 cells (ER+), T47D (PR+), SKBR-3 (HER2+), MDA-MB-231 (Ki67+), skin tissue (CK5/6+), and appendix tissue (vimentin+).

### 3.5. Antiproliferative Effects of Different Drugs in Canine Mammary Tumor Cell Lines

The susceptibility of the cell lines CMTF4, CMTP8, CMTK10, CMTN7, and CMTL9 to anticancer drugs DOX, 5-FU, colchicine, paclitaxel, and carboplatin was evaluated using the MTT assay. DOX, 5-FU and carboplatin are drugs most commonly used for the treatment of canine mammary tumors [63,64,65].

DOX showed the highest growth-inhibitory effect (DOX > Paclitaxel > Colchicine > 5-FU > Carboplatin). The cell line CMTF4 was sensitive to the effect of DOX (IC_50_: 4.37 ± 0.40 µM), paclitaxel (IC_50_: 0.04 ± 0.0003 µM), and colchicine (IC_50_: 0.19 ± 0.01 µM). In contrast, 5-FU (IC_50_: >50 µM) and carboplatin (IC_50_: 100 µM) demonstrated limited effect on these cells. The cell line CMTP8 was susceptible to DOX (IC_50_: <0.63 µM), paclitaxel (IC_50_: ≥10 µM), and colchicine (IC_50_: >50 µM). Carboplatin (IC_50_: >200 µM) and 5-FU (IC_50_: >200) had no effect at the doses evaluated. The CMTK10, CMTN7, and CMTL9 cell lines showed resistance to all drugs tested, as shown in Table 3.

## 4. Discussion

In this study, we successfully established and partially characterized ten canine mammary tumor cell lines derived from both primary tumors and metastatic sites. The tumorigenic potential of five lines (CMTF4, CMTG5, CMTN7, CMTP8, and CMTL9) was confirmed in vitro using the soft agar colony formation assay, where all of them demonstrated the ability to grow and form colonies in suspension. Six of the cell lines (CMTF4, CMTG5, CMTN7, CMTP8, CMTL9, and CMTK10) underwent ICC characterization. Of these, five cell lines were negative for ER, PR, and HER2 expression, classifying them as triple-negative; one cell line exhibited hormonal receptor positivity and was classified as luminal A according to the molecular breast cancer classification [10,59,66]. We further evaluated the sensitivity of these cell lines to commonly used breast cancer chemotherapeutics. DOX exhibited the highest antiproliferative effect in two cell lines, whereas 5-FU and carboplatin showed limited efficacy across the panel. The establishment and partial characterization of these canine mammary tumor-derived cell lines provide a valuable preclinical model for advancing our understanding of mammary tumor biology, improving diagnostic strategies, and informing therapeutic development for both canine and human breast cancer.

Mammary tumors represent the most frequent neoplasia diagnosed in non-neutered female dogs, and approximately 50% are malignant [2,67,68,69,70]. Canines are a useful animal model of comparative oncology, as they develop spontaneous mammary tumors that share a high degree of similarity with human breast cancers [70]. Several molecular features of canine mammary carcinoma, including variable ER expression status, cyclooxygenase overexpression, p53 mutations, among others, are very similar to those seen in humans [16,71,72,73]. Cell lines are the in vitro model most utilized to study processes involved with carcinogenesis, like proliferation, migration, and apoptosis, as well as in preclinical studies for developing and testing new drugs or products with antiproliferative effects [70,71].

To assess the anchorage-dependent growth potential of the generated cell lines, a soft agar colony formation assay was performed. This qualitative assay evaluates in vitro tumorigenicity by measuring the cell’s ability to proliferate and form colonies in suspension within an agarose matrix [50,51,52,74]. Five of the stablished cell lines (CMTG5, CMTP8, CMTF4, CMTN7, and CMTL9) demonstrated the capacity to grow and form colonies. All these cell lines, with the exception of CMTL9 (for which histopathological data were unavailable), were confirmed as mammary carcinomas. Notably, CMTG5, CMTP8, and CMTF4 originated from metastatic lesions. The human breast cancer cell line MCF7, derived from pleural effusion of woman with breast cancer with metastatic disease [75], served as a positive control, consistent with its previously documented colony-forming ability in suspension [76,77,78,79]. Anchorage-independent growth is closely associated with anoikis resistance, a hallmark of malignancy characterized by the evasion of apoptosis triggered by detachment from the extracellular matrix [80,81]. The ability of CMTG5, CMTP8, CMTF4, CMTN7, and CMTL9 to survive and proliferate under anchorage-independent conditions suggests a high degree of malignancy and metastatic potential, reinforcing their relevance as in vitro models for studying aggressive breast cancer phenotypes.

In women with breast cancer, tumors are generally classified into five molecular subtypes: luminal A, luminal B, luminal B with HER2, HER2-enriched, and triple-negative. This classification allows for the selection of specific targeted therapies, such as anti-estrogen drugs luminal subtypes and monoclonal antibody-based immunotherapy like trastuzumab for HER2-positive subtypes [82]. Similarly, studies on canines with malignant mammary tumors have applied analogous markers, revealing that tumors lacking ER and PR generally exhibit poorer outcomes, akin to triple-negative breast cancer in humans, which is characterized as an aggressive disease, with poor prognosis [83,84,85].

In canine studies, luminal A-type tumors, which typically have a better prognosis, are often associated with lower-grade malignancies and reduced lymphatic invasion. Luminal A is the most common subtype, accounting for 44% of cases in a sample of 159 canine mammary tumors [66]. The six canine cell lines assessed in this study were derived from mammary tumors, including three from metastatic sites, hinting at a potentially aggressive molecular profile. Notably, the CMTP8 cell line positive for hormone receptors, originating from a metastatic site, demonstrating that tumors with these characteristics can have the potential to invade and metastasize. This correlates with the positivity for vimentin and CK5/6, suggesting a potential basal-like or hybrid epithelial/mesenchymal phenotype. It could also reflect an intermediate state in the continuum of EMT.

Ki67 is a proliferation marker and is considered a prognostic factor in canine mammary tumors [69,73]. The antigen Ki67 encoded by the MKI67 gene plays a critical role in mitotic processes. It is a nuclear, non-histone protein expressed in dividing cells. Ki67 acts as a surfactant to maintain chromosome separation following nuclear envelope breakdown, contributes to the formation of the perichromosomal protein compartment through interaction with protein phosphatase 1, and facilitates chromosome binding to the mitotic spindle as well as their mobility. Moreover, Ki67 helps organize heterochromatin and excludes large cytoplasmic molecules, such as ribosomes, from nuclei reassembled at the end of mitosis [86]. Tumors with higher proliferation rates showed an increased expression of Ki67 [87]. In this study, the cell line CMTF4 exhibited positive Ki67 expression and had the fastest duplication times among the evaluated cell lines (22 h). This rapid proliferation could also be linked to the metastatic origin of the CMTF4 line. As for Ki67-negative cell lines, this could indicate the predominance of a quiescent or slow-cycling subpopulation within the culture, reflecting tumor heterogeneity. Alternatively, epigenetic or genetic modifications of the MKI67 gene, phenotypic changes during in vitro adaptation, or culture conditions could explain the absence of detectable Ki67, despite the malignancy of the cells. It has been shown that Ki67 depletion results in reduced proliferation [88]. Additionally, it should also be considered that the growth of cell lines is dependent on many factors like temperature, oxygen, nutrients, and pH, among others, to grow [47].

CK5/6, a myoepithelial or basal epithelial marker, was found to be positive in the CMTP8, CMTG5, and CMTK10 cell lines. CMTF4, CMTN7, and CMTL9 did not express this marker. Vimentin, a mesenchymal marker, was positive across all six cell lines CMTF4, CMTN7, CMTL9, CMTP8, CMTG5, and CMTK10. CK5/6 expression, typically associated with basal cytokeratins, combined with vimentin, highlights a degree of phenotypic plasticity. The overexpression of vimentin in tumors of epithelial origin has been associated with phenotypic changes such as EMT, a process where epithelial cells lose their typical characteristics and acquire a mesenchymal phenotype. These changes lead to increased cell mobility, invasiveness, and metastatic capabilities [89,90]. The widespread expression of vimentin across all cell lines further underscores the mesenchymal traits common in aggressive cancer phenotypes and carries poor prognosis, particularly in cases of triple-negative breast cancer [91]. Notably, the cell lines CMTF4, CMTN7, CMTL9, and CMTG5, which were classified as triple-negative, also demonstrated the ability to proliferate and grow independently of substrate attachment, as evidenced in the soft agar colony formation assay, underscoring their potentially high metastatic capacity.

We acknowledge the limitations of marker-based interpretation in the absence of a full panel; however, the combined cellular behavior and marker expression patterns in our cell lines are consistent with tumorigenic characteristics previously reported in canine mammary cancer models [92,93,94]. Therefore, our interpretation is carefully contextualized within this framework and does not overstate the implications of CK5/6 or vimentin expression alone.

The ICC profiles and morphological characteristics of the established canine mammary cancer cell lines highlight their intrinsic heterogeneity and malignant potential. These findings support their utility as biologically relevant in vitro models for studying canine mammary tumorigenesis, particularly the dynamic interplay between basal-like and mesenchymal phenotypes. Such models may also offer translational value for human breast cancer research, particularly in the context of aggressive subtypes and the identification of novel therapeutic targets. Future investigations should further dissect the molecular pathways underlying these dual phenotypes and assess their response to various therapeutic agents, thereby advancing our understanding of tumor progression and treatment resistance mechanisms.

Canine mammary tumor cell lines also serve as effective platforms for evaluating the antiproliferative effects of chemotherapeutic agents. In veterinary oncology, surgical excision remains the primary treatment for mammary tumors; however, high-grade or aggressive subtypes require adjuvant chemotherapy. Several agents including DOX, carboplatin, 5-FU, and mitoxantrone have been employed with varying degrees of success [63,64,65,67]. Nevertheless, comprehensive data on their efficacy and impact on survival outcomes in dogs remain scarce.

In the present study, we assessed the cytotoxic profiles of five compounds: DOX, paclitaxel, colchicine, 5-FU, and carboplatin. DOX, an anthracycline antibiotic that intercalates within DNA base pairs, inhibits DNA and RNA synthesis, and acts as a topoisomerase II inhibitor [95,96], exhibited the highest antiproliferative activity in two of the tested cell lines. This finding aligns with prior studies demonstrating its efficacy in both human and canine breast cancer models [63,97,98]. However, despite its widespread use, clinical data in dogs have shown that while DOX improves local tumor control, it does not significantly enhance overall survival [63]. In contrast, preclinical studies using two canine mammary cancer cell lines reported that DOX showed an inhibitory effect on cell proliferation in both cell lines [99]. These are similar to our results, where the cell lines CMTP8 and CMTF4 were susceptible, while displaying no effect on proliferation. This variable effect of the drugs may be due to the heterogenicity of breast cancer and could also be related to resistance mechanisms, such as increased activity of the ATP-binding cassette transporters, as reported in human breast cancer [100,101].

Paclitaxel is widely used for the treatment of breast cancer in women and belongs to the class of taxanes. It targets microtubules and induces apoptosis at G0 and G1/S phases of the cell cycle [102,103]. Paclitaxel has shown efficacy in affecting cell viability in canine tumoroids, similar to what has been observed in humans. In another study, with canine mammary tumor cells, paclitaxel inhibited proliferation, as well as inducing apoptosis and cell cycle arrest [104,105]. Similarly, as seen in CMTP8 and CMTF4 cells, paclitaxel inhibited their proliferation. In contrast, CMTK10, CMTN7, and CMTL9 cells were less sensitive to the effect of this drug. This difference in sensitivity could be associated with the fact that CMTP8 and CMTF4 cells are susceptible to the suppression of microtubule dynamics. For the rest of the evaluated cell lines, a possible resistant mechanism is active as described in human breast cancer, like the overexpression of TLR4 (toll-like receptor-4), overexpression of RNF8 (finger protein 8), and overexpression of HER2, among others [106,107].

Colchicine is a tubulin inhibitor with antiproliferative effects inducing mitotic arrest and cell death [108,109]. This compound was evaluated in breast cancer cell MCF7, demonstrating its antiproliferative capacity, thus inducing apoptosis [109]. Colchicine has limited application in patients due to its side effects and toxicity [110]. In this study, CMTF4 was sensitive to the effects of colchicine. The cell lines CMTP8, CMTK10, CMTN7, and CMTL9 were resistant to the antiproliferative effects of colchicine.

The 5-FU belongs to the antimetabolite class of chemotherapeutics, induces the inhibition of DNA and RNA synthesis, and inhibits thymidylate synthase [111,112]. The five cell lines evaluated showed no effects from this drug. Multiple factors could be associated with resistance to 5-FU, such as overexpression of Bcl-2, alteration of transport pathway, and overexpression of thymidylate synthase, among others [113,114].

Carboplatin is a platinum-based drug that inhibits DNA replication and transcription by forming DNA adducts. This drug is used as adjuvant chemotherapy for canine mammary cancer [64,115]. In the CMTF4, CMTP8, CMTK10, CMTN7, and CMTL9 cell lines, carboplatin showed no antiproliferative effect, which may be associated with increased efflux of the drug, enhanced DNA repair mechanisms, and the presence of apoptosis inhibitors, among other factors [115,116].

Our data indicate that the established cell lines express malignancy traits reflective of the primary mammary tumor or metastatic site, exhibiting varying responses to the antineoplastic drugs. Specifically, cell lines CMTF4 and CMTP8 showed sensitivity to DOX and paclitaxel, while the majority of drugs tested had minimal effects on CMTK10, CMTN7, and CMTL9. These cell lines, to the authors’ knowledge, are the first canine mammary tumor cell lines generated in Mexico and provide valuable models for breast cancer research—including studies on metastasis, EMT, and etiology of breast cancer—considering that canines can act as sentinels for human exposures to different elements that can be involved in carcinogenesis and for the development and evaluation of new treatments. Additionally, the use of canine mammary tumor cell lines should be considered in patient-specific therapies to improve treatment outcomes and survival rates.

## 5. Conclusions

Ten canine mammary tumor cell lines were established and partially characterized from spontaneous tumors of dogs living in Mexico, including both primary and metastatic sites. The cell lines exhibited diverse morphological and immunocytochemical profiles, including triple-negative and luminal A phenotypes, and several demonstrated in vitro tumorigenicity and differential drug sensitivity. These models represent a valuable tool for breast cancer research and will facilitate the evaluation of therapies or compounds with antiproliferative effects, aiming to improve treatment outcomes, quality of life, and survival in both canine and human patients with breast cancer.

## Figures and Tables

**Table 1:** Clinical data of canines with mammary tumor used for the establishment of cell lines.

Number of Cell Line	Name of the Cell Line	Age (Years)	Breed	Reproductive Status	Biological Sample	Histopathology	Grade
1	CMTM1	15	Chihuahueño	Spayed	Mammary tumor	Intraductal papillary adenoma	Benign tumor
2	CMTM11	12	Chihuahueño	Unspayed	Mammary tumor	Tubular adenoma with cellular dysplasia	Benign tumor
3	CMTP3	13	Mixed	Unspayed	Mammary tumor	Benign mixed tumor with intraductal papillary adenoma	Benign tumor
4	CMTL9	15	Pit bull	Spayed	Mammary tumor	ND	ND
5	CMTP6	11	Poodle	Spayed	Mammary tumor	Tubular carcinoma	I
6	CMTN7	15	Chihuahueño	Unspayed	Mammary tumor	Mixed-type adenocarcinoma	I
7	CMTK10	16	Poodle	Unspayed	Mammary tumor	Solid adenocarcinoma	II
8	CMTG5	13	Maltes	Spayed	Pleural effusion	Infiltrating tubulopapillary carcinoma	II
9	CMTP8	14	Mixed	Spayed	Pleural effusion	Intraductal carcinoma	I
10	CMTF4	12	Golden retriever	Spayed	Pleural effusion/lung metastatic nodule	Infiltrating intraductal papillary carcinoma	I

ND: Not determined.

**Table 2:** Expression of hormonal, epithelial, and mesenchymal markers on canine mammary cell lines.

	Cell Marker				Epithelial Marker CK5/6	Mesenchymal Marker Vimentin
Cell Line	ER	PR	HER2	Ki67		
CMTF4	−	−	−	+	−	+
CMTN7	−	−	−	−	−	+
CMTL9	−	−	−	−	−	+
CMTP8	+	+	−	−	+	+
CMTG5	−	−	−	−	+	+
CMTK10	ND	−	−	−	+	+

ER: Estrogen receptor, PR: Progesterone receptor, HER2: Human epidermal growth factor receptor 2, Ki67: Proliferation marker, CK5/6: Cytokeratin 5/6, ND: Not determined.

**Table 3:** Doxorubicin showed the highest antiproliferative effect in two cell lines. The effect of DOX, paclitaxel, colchicine, 5-FU, and carboplatin in canine mammary tumor cell lines was evaluated by MTT assay.

Drug	Cell Lines				
	CMTF4	CMTP8	CMTK10	CMTN7	CMTL9
	IC_50_ (µM)				
DOX	4.37 ± 0.40	<0.63	>50	>50	>50
Paclitaxel	0.04 ± 0.0003	≥10	>50	>100	>100
Colchicine	0.19 ± 0.01	>50	>100	>100	>100
5-FU	>50	>200	>50	>50	>50
Carboplatin	>100	>200	>200	>200	>200

The values of IC_50_ represent the minimal concentration of a drug that is required for 50% inhibition in vitro. IC_50_ represents the media calculated from the results of three independent MTT experiments. DOX: Doxorubicin, 5-FU: 5-Fluorouracil.

## Data Availability

Data available on request from the authors.
